# Tailoring International Pressure Ulcer Prevention Guidelines for Nigeria: A Knowledge Translation Study Protocol

**DOI:** 10.3390/healthcare3030619

**Published:** 2015-07-28

**Authors:** Rose Ekama Ilesanmi, Brigid M. Gillespie, Prisca Olabisi Adejumo, Wendy Chaboyer

**Affiliations:** 1 Department of Nursing, College of Medicine, University of Ibadan, Ibadan 23402, Nigeria; E-Mail: bisiandbayo@yahoo.com; 2 NHMRC Centre of Research Excellence in Nursing, Centre for Health Practice Innovation, Menzies Health Institute Queensland, Griffith University, Gold Coast, Queensland 4222, Australia; E-Mails: b.gillespie@griffith.edu.au (B.M.G.); w.chaboyer@griffith.edu.au (W.C.)

**Keywords:** pressure injury, care bundle, knowledge translation, less developed economies

## Abstract

*Background*: The 2014 International Pressure Ulcer Prevention (PUP) Clinical Practice Guidelines (CPG) provides the most current evidence based strategies to prevent Pressure Ulcer (PU). The evidence upon which these guidelines have been developed has predominantly been generated from research conducted in developed countries. Some of these guidelines may not be feasible in developing countries due to structural and resource issues; therefore there is a need to adapt these guidelines to the context thus making it culturally acceptable. *Aim*: To present a protocol detailing the tailoring of international PUPCPG into a care bundle for the Nigerian context. *Methods*: Guided by the Knowledge to Action (KTA) framework, a two phased study will be undertaken. In Phase 1, the Delphi technique with stakeholder leaders will be used to review the current PUPCPG, identifying core strategies that are feasible to be adopted in Nigeria. These core strategies will become components of a PUP care bundle. In Phase 2, key stakeholder interviews will be used to identify the barriers, facilitators and potential implementation strategies to promote uptake of the PUP care bundle. *Results*: A PUP care bundle, with three to eight components is expected to be developed from Phase 1. Implementation strategies to promote adoption of the PUP care bundle into clinical practice in selected Nigerian hospitals, is expected to result from Phase 2. Engagement of key stakeholders and consumers in the project should promote successful implementation and translate into better patient care. *Conclusion*: Using KTA, a knowledge translation framework, to guide the implementation of PUPCPG will enhance the likelihood of successful adoption in clinical practice. In implementing a PUP care bundle, developing countries face a number of challenges such as the feasibility of its components and the required resources.

## 1. Introduction

International clinical practice guidelines (CPG) for pressure ulcer prevention (PUP) provide guidance to improve the uptake of prevention strategies, reduce unwarranted variation in care and ultimately improve patient outcomes [1]. But, a number of unique barriers related to feasibility and resources exist in implementing guidelines in developing countries. This paper presents a protocol to tailor international pressure ulcer prevention clinical practice guidelines (PUPCPG) to the Nigerian context. By using the Knowledge to Action (KTA) framework [2] and developing a structured process to prepare for PUPCPG uptake, the processes detailed in this protocol may be used as a guide by others, aiming to improve a range of other clinical practices in developing countries.

CPGs are systematically developed evidence-based statements to assist practitioners and patients’ decisions about appropriate health care for specific circumstances [3,4]. The statements are based on standardized best practices, and have shown to support improvements in quality and consistency in healthcare, as well as reduce inappropriate variations in practice [5,6]. High quality CPGs are based on a thorough evaluation of evidence from published research studies on the outcomes of treatment or other health care procedures.

The 2014 internationally developed PUPCPG (National Pressure Ulcer Advisory Panel(NPUAP), European Pressure Ulcer Advisory Panel (EPUAP) and Pan Pacific Pressure Injury Alliance (PPPIA)) [7] target eight prevention strategies including assessment of risk factors for PU, skin and tissue assessment, preventive skin care, nutritional assessment and provision of optimal nutrition, repositioning and early mobilization and the use of support surfaces. This new document presents a compendium of a large body of evidence for PU prevention, which if effectively integrated into practice, will enhance clinical outcomes. Wallin [8] claims the persistent high prevalence of hospital acquired PU (HAPU) stems from inadequate compliance with existing CPGs (*i.e*., inadequate knowledge translation). Consequently, patients continue to experience unnecessary suffering, prolonged hospitalization and continued ongoing treatment with financial burden to the patient and the health care system [8].

As a consequence of the complications associated with PUs, in 2006 the Institute of Healthcare Improvement (IHI) included PUP as one of the 12 interventions required to achieve the campaign goal of saving five million lives from iatrogenic harm [9]. This also further emphasizes the significance of knowledge translation in PU prevention through consistent application of CPGs, in order to close the evidence-practice gap. Importantly, there is a growing body of evidence to support the claim that implementing PUPCPGs will improve patients’ care outcomes [10,11,12]. Yet, in reality we know that implementing CPGs in clinical practice is suboptimal and variable [13]. However, uptake of guidelines, with resultant improvements in professional practice is possible when interventions underpinned by specific guidelines are tailored to address identified barriers [14].

Over the past decade or more, a care bundle approach has been advocated as one tool to promote CPG uptake [9]. “Care bundles” are a set of evidence-based recommendations for a defined patient segment/population and care setting that, when implemented together, will result in significantly better outcomes than when interventions are implemented individually [15,16]. They are tools to assist with implementing CPGs into practice. The bundle approach was first introduced by IHI in 2001, and at that time it was focused on providing safe and reliable care to patients on ventilators and those with central lines in the Intensive Care Units (ICU) [17]. Each of those bundles consisted of a set of four to five evidenced-based interventions, respectively [17,18]. The bundle approach has produced significant improvements in patient care over non-structured approaches [19]. Some examples where care bundles have been used in clinical practice with evidence of benefits include; ventilator care bundle [20], routine care of intensive care patient [16], and maintaining central lines in paediatric populations [21,22]. In a recent study, Chaboyer and colleagues have developed a care bundle aimed at encouraging active patient participation in PUP with promising results [23,24].

## 2. Conceptual Framework

The proposed protocol is underpinned by the KTA framework [2]. It provides a structure to facilitate the uptake of CPGs into clinical practice. This framework has both a knowledge generation and an action cycle, and it is this action cycle that focuses on implementation issues. The activities within the action cycle include: (1) adapting knowledge to local context; (2) assessing barriers and facilitators; (3) involving stakeholders; (4) tailoring knowledge to the needs of the potential users; (5) monitoring knowledge use; (6) evaluating outcome; and (7) mechanism to sustain knowledge use. This study focuses on the first four activities within the action cycle of the KTA framework using qualitative research method.

Study Goals:

1. To identify a small set of PU prevention interventions (likely three to eight) from recommended CPG that are feasible to implement in Nigeria, to comprise a PUPCB.
2. To explore contextual factors that may act as facilitators or barriers for implementing the PUPCB.

## 3. Study Design/Methodology

A two-phased knowledge translation study will be undertaken as shown in Table 1. In Phase 1, the study will focus on tailoring knowledge to the local context (KTA activity 1). This will subsequently guide the assessment of the feasibility of using the current PUPCPGs in selected acute care teaching hospitals in Nigeria. In Phase 2, facilitators, barriers and strategies to promote successful adoption will be identified (KTA activity 2). Key stakeholders will be involved in tailoring the PUP CPGs into a care bundle and in devising strategies for its implementation in the Nigerian hospital context (KTA activities 3 and 4).

**Table 1:** Duration of Project/Time Frame.

Months 1–4 January–April 2016	Months 5–8 May–August 2016	Months 9–10 September–October 2016
Phase 1: Delphi study	Phase 2: Interview and Draft Manuscript	Report Writing and Disseminating Results

### 3.1. Phase 1

Design: A quantitative method using the Delphi technique will be used to identify three to eight PU prevention interventions from recommended CPGs that are feasible in Nigeria, to comprise a PUPCB. The Delphi process has been described as a structured process whereby a panel of informed individuals or “experts”, through a process of repeated questions and feedback reach a consensus on a particular issue [25,26]. In the IHI document on care bundles, Resar *et al.* [16] suggested that, for effective application, all elements included in any care bundle must be based on the consensus of the local clinicians (*i.e*., stakeholders). Others advocate wider end-user input such as patients and families [24]. We anticipate the Delphi process [25]; will generate a consensus on the implementation of three to eight evidence-based interventions that are feasible, which will then become the PUPCB.

Setting and Sample: A total of sixty-four (64) experts: nurses, doctors and healthcare consumers will be selected from eight teaching hospitals in the South West and North Central geo-political zones of Nigeria. In each hospital, the Director of clinical nursing; a management decision maker, and two other senior nurses who have a background and experience in PU prevention, to be nominated by the Nursing director will be selected. The medical expert will include the Chairman, of the Medical Advisory Committee; also a management decision maker, and other two doctors who also possess background and experience in PU prevention and management, to be nominated by the chair. Selection of the expert panel is based on their roles either as management decision makers or those with a experience in PU prevention in their respective hospitals in the selected zones of the study. These professional experts in each hospital will nominate two healthcare consumers to be included. In all, eight experts from eight hospitals will constitute a panel, making a total of 64 respondents. The constitution of the expert committee membership can serve as a prototype model for hospitals in other zones of the country. While this setting does not include all of Nigeria, it does reflect all teaching hospitals in a large region, with a large sample capturing a diversity of opinions.

PUP Questionnaire: The PUP questionnaire that will be used in the Delphi rounds will be based on the international CPG [7]. The questionnaire (Supplementary 1) will consist of eight subsections, including:

Risk assessment:

1. Skin assessment
2. Preventive skin care practices
3. Nutritional assessment and PU prevention
4. Standards for repositioning and early mobilization
5. Positioning devices
6. Support surfaces
7. Patient and caregiver participation

A 4-point response scale from 1 to 4 “very easy”, “somewhat easy”, “somewhat difficult”, and “very difficult/impossible to implement” will be used. For responses “somewhat difficult” and “very difficult/impossible to implement”, respondents will be asked to describe what they think the implementation barriers are.

The questionnaire will be piloted in a federal medical centre, with similar facilities and patient acuity, as obtained in the hospitals for the actual study. The Federal medical centre is a federal government funded public hospital, like the teaching hospitals. The questionnaire will be in English language, which is the official language in Nigeria.

Delphi process:

1. Letters to the heads of each hospital will be sent to inform them of the Delphi process. They will be asked to nominate medical and nursing representatives who are directly involved in PU prevention as well as healthcare consumers/patient representatives to the panel.
2. An electronic survey using the SurveyMonkey^©^ (Palo Alto, California) program will be developed and sent to the panel members. Responses will be received electronically.
3. Respondents will be asked to rate the feasibility of each intervention and to describe what they think the implementation barriers are.
4. Responses will be expected within two weeks after which, at least two reminders will be sent.

For each item, 75% agreement will be considered a consensus.

Data Analysis: The four point response scale (1–4) “very easy”, “somewhat easy”, “somewhat difficult”, and “very difficult/impossible” will be recoded from 4 responses to 2. Once this recoding from four to two responses occurs, we will then calculate the frequency of each grouped response option. Then, we will require a relative frequency (*i.e*., percent) of 75% or more of the recoded response option of “easy to implement” for the item to be retained for the second round. Responses obtained in round 2 will be similarly analyzed as in round 1 and those items that receive a 75% consensus in the second round will become the components of the PUP care bundle, which we anticipate will be feasible and contextually appropriate.

### 3.2. Phase 2

Design: Interviews with key stakeholders will explore contextual factors that may pose as facilitators and/or barriers to implementing the PUPCB. This phase represents activities 2 to 4 in the KTA action cycle.

Setting and Sample: A purposive sample of key stakeholders including doctors and nurses in various roles (directors, managers, educators and bedside nurses) and healthcare consumers will be recruited to the study. They will be drawn from the same hospitals as in Phase 1. We anticipate recruiting 20 stakeholder participants, but recruitment will continue until data saturation is reached (*i.e*., no new ideas emerging from the interviews). Many stakeholders will be the leaders and policy makers in each hospital. Aarons *et al.* [27] reports the impact of tangible organizational support in adoption, implementation and sustenance of evidence-based interventions.

Data Collection: Face to face, semi-structured recorded individual interviews will be conducted. However, where this is not feasible, telephone interviews will be organized. The interview guide (Supplementary 2) will focus on facilitators, barriers and implementation strategies and will be based on the Theoretical Domains Framework (TDF) [28]. The domains of the TDF target aspects of individual behavior related to knowledge/skills, capacity (*i.e*., self-efficacy), social influences, beliefs, personal motivations and goals, decision-making and practice context. For example, to ascertain whether *knowledge* is a barrier or an enabler, key stakeholders may be asked the following question: “*What is your understanding of the PUPCB?*” A question that addresses the TDF domain of *beliefs about capacity* may include “*How easy or difficult is it to use the PUPCB?*” To establish whether the domain of *personal motivations and goals* act as either a barrier or enabler might be, “*Can you describe your perceptions of the extent to which pressure ulcer prevention is a priority in your hospital?*”

Data Analysis: All interviews will be transcribed and read through repeatedly in order to make sense of the data [29]. The process of data preparation will involve organizing the data to identify the unit of analysis. Both deductive and inductive content analysis approaches will be used. In the deductive approach, a categorization matrix will be developed to sort the data into three main domains: barriers, facilitators and stategies. This will be followed by an inductive approach for each domain [29]. Inductive analysis involves reading and rereading the data, coding the data and then grouping the coded data into subcategories and then categories, and giving each a thematic label.

A number of steps will be undertaken to promote the rigor of analysis, such as ensuring the credibility and dependability of the data [30,31]. To achieve this goal, items used for semi structured interview will be underpinned by the TDF framework, and the data sources will be triangulated by using a wide range of informants, to provide a rich source of information [32]. We will conduct “on the spot” and “end of dialogue” member checks, to verify individual viewpoints and ascertain the accuracy of the data. Guba and Lincoln [30,31] described member checks as an important method of ensuring the trustworthiness and credibility of qualitative data. We will import our interview data into the Nvivo software, version 10 [33], to create free nodes (themes), into which related themes will be organized. This will allow for consistent coding schemes and provide a robust interpretation of the data [34]. Furthermore, we will keep an audit trail, demonstrating our analytic decisions and showing how the findings are grounded in the data [35]. This is to further ascertain the credibility of the interview data. In addition, the research team will hold regular debriefing meetings to discuss the emerging findings.

## 4. Expected Results

PUP care bundle, with three to eight components is expected to be developed from Phase 1. Implementation strategies to promote adoption of the PUP care bundle into clinical practice in selected Nigerian hospitals is expected to result from Phase 2. Engagement of key stakeholders and consumers in the project should promote successful implementation and translate into better patient care.

## 5. The Clinical Implications

The global emphasis on evidence-based, cost effective and accountable healthcare has stimulated increasing interest on how to transfer the outcomes of rigorous research evidence (knowledge) to practice. Yet, there is documented evidence suggesting that research findings are slowly translated into practice, despite huge investments into healthcare research [2,36]. As a result, patients are not receiving the best care possible. Researchers in the science of knowledge transfer argue that the evidence-practice gap, *i.e.*, inadequate translation of research evidence into practice invariably results in poor health outcomes, where patients fail to benefit optimally from advances in health care. Consequently poorer quality healthcare and loss of productivity ensue [37]. It follows therefore that effective translation of research into practice is beneficial to patient care and clinical outcomes.

Traditional approaches to incorporating evidence based guidelines into clinical practice have focussed on quality improvement initiatives that are not necessarily theory-driven. As such, it is difficult to understand the mechanism of “success” or “failure” during the implementation cycle [38]. Clearly, embedding the use of CPG into practice essentially relies on behavior change. Yet, changing clinicians’ practice or behavior is reportedly difficult in general. Some authors propose the use of learning theories, which suggest that a particular behavior will likely be repeated if it is associated with incentives [39], therefore reinforcement strategies such feedback and audits are promoted. These strategies, though promising have not successfully provided the desired behavioral change in clinical practice. Undoubtedly, implementation interventions that are theory driven have proved successful in achieving behavior change in clinical practice [40,41]. One of such theories is the KTA framework.

In our study, we will use the KTA framework as a foundation coupled with the TDF [28], to theoretically drive the planned implementation of PUP evidence into practice. As many health care outcomes are influenced by health professionals’ behavior [42], implementation strategies must be specifically tailored to target behavior and take into account the practice context. The KTA framework assumes a systems perspective, which holds that the healthcare system is a subsystem within the social system, and is responsive and adaptive in both predictable and non-predictable ways [2]. The KTA is therefore a dynamic, iterative, and complex synthesis of knowledge creation and application.

Components of our implementation of the PUPCB in the Nigerian context will be guided by the TDF framework, as described by Michie and colleagues [28]. This framework allows us to subsequently develop implementation interventions that are specifically targeted to changing clinicians’ behaviors based on identified contextual barriers and enablers. Behavior change is central to effective clinical practice and ensures quality care [43]. By definition, behavior change interventions are a coordinated set of strategies specially developed to change particular patterns of behavior [43]. For sustained behavior change, it is essential that the development of such interventions is grounded in evidence. Therefore understanding the nature of the behavior that requires change, the characteristics of the practitioners involved, and the context is important.

Nigeria is a country with limited resources and most of the evidence used in the development of the PUPCPG has emanated from studies conducted in developed countries. Based on this understanding, it is imperative to tailor these PUPCPG to the Nigerian context. This current study has a potential to benefit care consumers and to assist healthcare providers to make better use of existing knowledge within the context of limited resources. Implicitly, evidence based nursing practice is an integration of the best evidence available, nursing expertise, values and preferences of the individuals, families and communities which they serve [44]. Contextual influences such as patients’ status and available resources in the organization must also be considered. This proposed study adopts a rigorous methodology that will effectively position the integration of evidence within the local context. Variability in organizations such as decision making processes, infrastructure, size, degree of specialization, *etc*., may influence uptake of research evidence [38]. Thus for successful uptake and dissemination of evidence, a comprehensive understanding of the organisation is imperative. In our study, the key stakeholder interviews will be conducted with decision makers and end users, who will identify the possible barriers and facilitators as well as strategies to implementing PUPCB in each care facility. Triangulation of data from a wide range of informants (doctors, nurses and patients) will enable a more nuanced understanding of the barriers and strategies, and what elements are more likely to be feasibly tailored in the PUPCB. This study will present opportunities for further research and next level of actual implementation and evaluation of the effectiveness of the PUPCB among at-risk patients in Nigerian hospitals, because the developed PUPCB will be an easy-to use care tool that will reduce PU incidence among patients in Nigeria. All these constitutes the strength of the study.

## 6. Limitations of Research Methods/Problems Anticipated

We anticipate some challenges because the Delphi process is time consuming, being an iterative process and may delay the process of data analysis and development of revisions based on prior responses. In view of this, the investigators will send reminders to prospective respondents during the process. It is hoped this approach will facilitate responses. The study will be conducted in only two of six geopolitical zones in Nigeria, which means the findings may not reflect other hospitals or zones. It is, however, believed that the findings will provide insights that are applicable to other settings.

## 7. Ethics and Informed Consents

The study proposal will be submitted to the Institututional Review Boards (IRB) of each teaching hospital for approval. The study will adhere to the international ethical guidelines with participants having the right to refuse participation or withdraw at any stage. There are no anticipated risk to the aprticipants.

## 8. Conclusions

The international CPG provide the most up to date recommendations for PUP. However, the feasibility of their use in developing countries, such as Nigeria where resource constraints is an everyday reality is unknown. Using the KTA framework to underpin investigation of the applicability of these PUPCPGs to the developing world will allow adaptation of the CPGs into a practical, user friendly care bundle. Understanding the barriers and facilitators to adopting this care bundle, will assist with its implementation. The protocol proposed detailed herein can be used as a template for others to adapt guidelines to developing countries, where resource constraint is a common phenomenon.

## Supplementary Files

Supplementary File 1
