# Supplementary material for: Tailoring International Pressure Ulcer Prevention Guidelines for Nigeria: A Knowledge Translation Study Protocol
Source: Healthcare (Basel). 2015 Jul 28;3(3):619–29. doi: 10.3390/healthcare3030619 (PMC4939576; doi:10.3390/healthcare3030619)
Supplement: Supplementary File 1 [file healthcare-03-00619-s001.docx]

Supplementary Materials

Supplementary 1: Questionnaire for Delphi Process

Pressure ulcer (PU) prevention is a patient safety and quality care issue that affects hospitalized patients. Currently there is no formal protocol for PU prevention in Nigeria, resulting in variability
in practices.

You are invited to participate in a panel to assess the feasibility of introducing international PU prevention guidelines in the Nigerian context using the Delphi process. The aim of participation in this expert panel is to obtain your opinion on PU prevention interventions that can be feasibly implemented in Nigeria. This is the first round of the Delphi technique. You will receive a feedback on grouped responses to this first round, and you will have the opportunity to participate in a second round. Your contribution to this initiative is very significant to the success of this project.

The aim of this study is to adapt the interventions recommended by international pressure ulcer prevention guidelines to develop a Pressure Ulcer prevention Care Bundle (PUPCB) that will be feasible to introduce in the Nigeria context.

Phase 1 of this study is a Delphi process and phase 2 will be a key informant interview.

As expert healthcare practitioners, we invite you to anonymously participate in a Delphi panel as part of the first round evaluation of the interventions to prevent pressure ulcer.

Section A this form asks for demographic information in relation to your hospital, professional practice and years of experience.

Section B of the form contains the specific recommended interventions to prevent PU.

We value having your input in developing this checklist and would appreciate completed surveys being returned by Wednesday 3rd April 2016. This is an online survey. Please return your completed survey to the researcher through the same email address.

We appreciate that you are a busy professional, and would be most grateful for your time and your expertise. If you have any questions, please feel free to contact one of the team members in your clinical area:

Please contact me if you have any questions about this project. On this email: *ekamailesanmi@yahoo.com.*

The project is funded by ________________________________.

The questionnaire will take about 30 minutes of your time. We thank you for participating in this research.

Rose Ilesanmi, RN, PhD.

Principal Investigator.

Section A: Demographic data

1. Name of Hospital________________________________
2. Please tick as Appropriate: Nurse ( ) Physician ( ) Healthcare Consumer ( )
3. Age ( years)
4. Years of Experience as Healthcare provider ( )

Section B: Assessment of Risks for PU

Instructions. The items listed below (Table S1) are evidence-based recommendations which have proved effective in preventing pressure ulcer. The goal of this study is to examine how feasible each of these interventions will be in Nigeria. Please indicate your response in relation to the extent to which the following pressure ulcer prevention strategies are easy/ feasible to implement in your healthcare context. If you chose “very difficult to implement or impossible to implement” please describe briefly what might be the barriers.

**Table S1.** Evidence-based recommendations for PU prevention.

| **Items** | **Very easy (1)** | **Somewhat easy (2)** | **Somewhat difficult (3)** | **Very difficult/ impossible (4)** |
| --- | --- | --- | --- | --- |
| **1. Risk Assessment** | 1 | 2 | 3 | 4 |
| A PU risk assessment should be performed within 8hours of admission. Repeated daily in patients at risk and weekly in those not at risk |  |  |  |  |
| A risk assessment tool should be adapted for use eg, Waterlow, Braden |  |  |  |  |
| **2. Skin assessment** |  |  |  |  |
| Inspect skin for localized heat, edema, induration, pressure ulceration, consistency in relation to surrounding tissue |  |  |  |  |
| Avoid skin massage over painful bony prominences |  |  |  |  |
| Apply moisturizing cream to dry skin as appropriate? |  |  |  |  |
| Inspect skin around or under medical devices at least twice daily for signs of pressure-related injury |  |  |  |  |
| **3. Preventive skin care practices** |  |  |  |  |
| Do not position patients on areas of erythema |  |  |  |  |
| Do not massage or rub at risk skin vigorously |  |  |  |  |
| Clean the skin promptly after each episode of incontinence |  |  |  |  |
| Use a barrier cream to protect the skin from excessive moisture |  |  |  |  |
| **4. Nutritional assessment and PU prevention** |  |  |  |  |
| Nutritional assessment for each patient at risk or with a PU |  |  |  |  |
| A valid nutritional screening tool should be incorporated into assessment |  |  |  |  |
| Develop an individualized nutritional plan for patients at risk for PU |  |  |  |  |

**Table S1.** *Cont.*

| **Items** | **Very easy (1)** | | **Somewhat easy (2)** | | **Somewhat difficult (3)** | | **Very difficult/ impossible (4)** | |  |
| --- | --- | --- | --- | --- | --- | --- | --- | --- | --- |
| **4. Nutritional assessment and PU prevention** | |  | |  | |  | |  | |
| Offer fortified foods with high calorie, high nutrition and supplements between meals | |  | |  | |  | |  | |
| Monitor for signs of dehydration: change in weight, skin turgor, urine output, elevated serum sodium, and serum osmolality | |  | |  | |  | |  | |
| Provide additional fluids for patients with heavily exuding wounds, profuse sweating, diarrhoea | |  | |  | |  | |  | |
| **5. Standards for Repositioning and early mobilization** | |  | |  | |  | |  | |
| Teach patients to do “pressure relief lifts” | |  | |  | |  | |  | |
| Avoid positioning individuals on bony prominences with existing non-blanchable erythema | |  | |  | |  | |  | |
| Use manual handling aids to reduce friction and shear: Lift and do not drag while repositioning | |  | |  | |  | |  | |
| Avoid positioning directly on medical devices e.g., tubes, drainage systems or other foreign objects. | |  | |  | |  | |  | |
| Do not leave patient on a bedpan longer than necessary | |  | |  | |  | |  | |
| Use 30° tilted side-lying position(alternately, right side, back, left side) or prone if individual can tolerate and medical condition allows | |  | |  | |  | |  | |
| Encourage patients who can reposition themselves to sleep on 30° to 40° side lying or flat if not contraindicated | |  | |  | |  | |  | |
| Avoid postures that increase pressure such as 90° side lying or semi-recumbent position | |  | |  | |  | |  | |
| Limit head-of-bed elevation to 30° for an individual on bed rest unless contraindicated by medical condition or feeding and digestive considerations. | |  | |  | |  | |  | |
| Avoid head-of-bed elevation or slouched position in a sitting position to prevent pressure and shear on the sacrum | |  | |  | |  | |  | |
| Use pressure redistribution surface while in prone position | |  | |  | |  | |  | |
| For sitting patients, provide adequate seat tilt to prevent sliding forward in a wheelchair | |  | |  | |  | |  | |
| Continue to turn and reposition regardless of support surface in use | |  | |  | |  | |  | |

**Table S1.** *Cont*.

| **Items** | **Very easy (1)** | | **Somewhat easy (2)** | | **Somewhat Difficult (3)** | | **Very difficult/ impossible (4)** | |  |
| --- | --- | --- | --- | --- | --- | --- | --- | --- | --- |
| **6. Positioning Devices** | |  | |  | |  | |  | |
| Do not use ring or dough-nut devices | |  | |  | |  | |  | |
| Do not use cut-out, ring donut-type devices to elevate the heels | |  | |  | |  | |  | |
| **7. Positioning Devices** | |  | |  | |  | |  | |
| Avoid using intravenous fluid bags to elevate the heels | |  | |  | |  | |  | |
| Avoid using water-filled gloves to elevate the heels | |  | |  | |  | |  | |
| Document repositioning regimes, specify frequency and position adopted and evaluation of outcome. | |  | |  | |  | |  | |
| Increase activity as rapidly as tolerated | |  | |  | |  | |  | |
| **8. Support surfaces** | |  | |  | |  | |  | |
| Avoid using small air cell (<10 cm) alternating mattresses or overlays | |  | |  | |  | |  | |
| Use an active support surface (overlay or mattress) for individuals at higher risk of PU when frequent manual repositioning is not possible. | |  | |  | |  | |  | |
| For patients with an existing PU, select pressure support surfaces that enhance redistribution, shear reduction and control of micro climate: beds with air fluidized features, beds with low air loss features, mattresses and overlays with alternating pressure features. | |  | |  | |  | |  | |
| **9. Incorporating patients and caregivers into PU prevention** | |  | |  | |  | |  | |
| Involve patient and care givers in all preventive strategies through education and pamphlets | |  | |  | |  | |  | |

Section C: Opinion on Possible Barriers to Implementing Evidence-Based Recommendations

Please comment freely about the barriers to introducing/using any of the above PUP strategies.

**____________________________________________________________________________________________________________________________________________________________________________________________________________________________________________________________________________________________________________________________________________________________________________________________________________________________________________________________________________________________________________**

Supplementary 2: Interview Guide

The interview is guided by the Theoretical Domain Framework (TDF), and it is aimed at identifying potential barriers, and facilitators of implementing PUPCB. These are draft questions from 5 behavioral domains of the TDF and will be refined based on the outcome of phase I.

Behavioral Change domains

1. Knowledge

Can you describe the strategies that are recommended for pressure ulcer prevention?

2. Beliefs about capabilities

What problems might be encountered in implementing pressure ulcer strategies? What strategies can help to increase use of PUPCB by nurses?

3. Beliefs and consequences

What in your opinion will be the short term and long term outcome for integrating the pressure ulcer prevention strategies into patient care?

4. Motivation and Goals

Can you describe your perceptions of the extent to which pressure ulcer prevention is a priority in your hospital?

5. Environmental context and resources

What resources are available for PU prevention in the hospital?

Can you think of barriers to implementing pressure ulcer strategies in your hospital?
